# Unravelling the conundrums of social autopsy for maternal mortality in low- and middle-income countries

**DOI:** 10.1371/journal.pgph.0004295

**Published:** 2025-03-06

**Authors:** Aduragbemi Banke-Thomas, Adedoyin Ogunyemi, Adeyemi Okunowo, Ololade Wright, Celso Monjane, Fatimat M. Akinlusi, Brenda Isikekpei, Phillip Wanduru, Rachel A. Thompson, Ndubuisi Ezumezu, Bosede B. Afolabi

**Affiliations:** 1 Faculty of Epidemiology and Population Health, London School of Hygiene & Tropical Medicine, London, United Kingdom; 2 Centre for Clinical Trials, Research, and Implementation Science, College of Medicine University of Lagos, Lagos, Nigeria; 3 Department of Community Health and Primary Care, College of Medicine. University of Lagos, Lagos, Nigeria; 4 Department of Obstetrics & Gynaecology, College of Medicine University of Lagos, Lagos, Nigeria; 5 Department of Community Health and Primary Health Care, Lagos State University College of Medicine, Lagos, Nigeria; 6 Instituto Nacional de Saude, Maputo, Mozambique; 7 Department of Obstetrics and Gynaecology, Lagos State University College of Medicine, Lagos, Nigeria; 8 School of Public Health, Makerere University College of Health Sciences, Kampala, Uganda; 9 Department of Global Public Health, Karolinska Institutet, Stockholm, Sweden; PLOS: Public Library of Science, UNITED STATES OF AMERICA

## Background

Any meaningful effort to tackle maternal mortality in low- and middle-income countries (LMICs) needs to be founded on a robust understanding of its causes. In LMICs, verbal autopsies are used to determine the clinical causes of death through interviews with the deceased person’s next-of-kin or caregivers. In addition, social autopsies have been conducted to allow capture of the story behind the death, enabling a comprehensive understanding of the socio-cultural, behavioural, and health system factors that contributed to the death [1,2]. However, researchers implementing social autopsies in LMICs are faced with some conundrums. In this opinion, we discuss three key conundrums and propose a way forward for the field.

## First conundrum: specific activities entailed and who to involve

Many researchers who have implemented social autopsies for maternal deaths have done so as an enquiry, i.e., a data collection process to understand the social factors contributing to the deaths [3]. However, others have described it as *“a group interaction with the family of the deceased woman and her wider local community”*, *“a potential health-promotion tool”* [4], and *“a tool for community dialogue”* [5]. These researchers engaged the deceased’s family members and neighbours to rehash questions aimed at exploring the *“social causes and errors behind the deaths*” in the presence of 40–50 individuals, including community leaders, teachers, local government personnel and health workers [5,6]. More recent guidance suggests that 30–50 persons, including the deceased’s family relatives, neighbours, local politicians, community leaders, and health facility representatives are needed for social autopsies [7].

With these approaches that conduct community dialogues as part of social autopsies, even if consent is obtained from relatives and names anonymised, it is highly likely that in small communities where everyone knows each other and with the relative rarity of maternal deaths, many people will know the deceased pregnant woman and her family. This situation will inadvertently fester blame and stigma, which the field wants to avoid [8]. In one study, despite the efforts of the dialogue facilitator to refocus the discussion, community members still blamed the health workers [6]. Indeed, there is an ethical question of whether the anticipated benefit of this additional community dialogue step outweighs its risks. The research may end, but the blame and stigma emanating from what essentially constitutes a ‘public law court’ to explore social causes of maternal deaths will outlive the project and many of those affected cannot speak for themselves. There is also the fundamental question of what actions the community can realistically finance and implement on their own to reduce maternal deaths. Actions such as promoting better health-seeking behaviour have been highlighted as outputs of community dialogue, but do community members need to be exposed to the details of the demise of a pregnant woman who lived amongst them before becoming such advocates of such interventions? Even when evidence from social autopsies reaches policymakers and service planners who can implement policy, there is little documentation of changes or improvements afterwards [8].

## Second conundrum: quantitative or qualitative? What tool to use?

For those who have implemented social autopsy as an enquiry, most have conducted or published them as quantitative surveys only, and only few have conducted qualitative studies or mixed methods. This is despite some of the available tools having qualitative questions [1,3]. There were three different tools identified from published social autopsy studies as per a 2017 review [3]. Another tool, consisting of quantitative and qualitative supplements has since been published [9]. For verbal autopsies, there is no confusion about its quantitative nature [10]. For social autopsies, which essentially seek to capture the stories and understand the ‘why’ behind the causes of deaths, it certainly makes methodological sense as to why qualitative data should be prioritised. Available tools have also been described as long, repetitive, especially when done separately from a verbal autopsy, and burdensome [11].

## Third conundrum: scale of implementation

The social factors contributing to death which social autopsies aim to explain are context-specific. As such, it is vital to consider the geographical scale at which social autopsies can generate a meaningful cohesive understanding of such factors. Previous studies have been conducted at different scales, from multiple facilities to across districts [3]. A typical community in LMICs will be a village, ward or sub-district. Even at this level, the population in the community might not be homogeneous enough to have similar factors contributing to maternal mortality or for collective action to be effective in fully tackling the public health issue.

## Way forward

**Fig 1:**
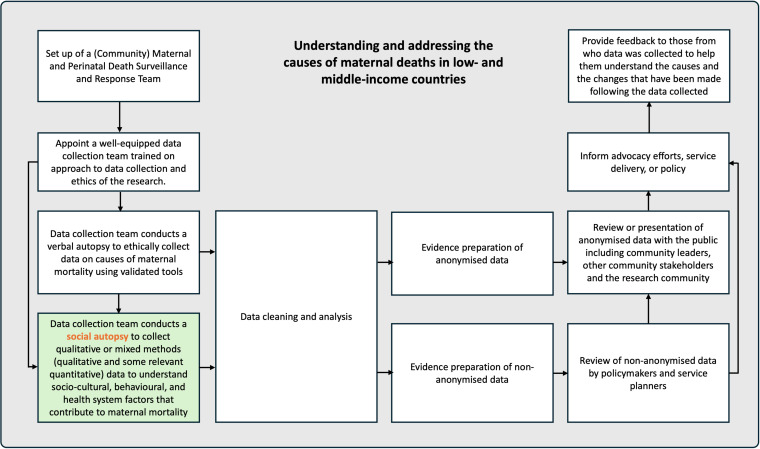
Social autopsy (highlighted as a box in green) within the broader ecosystem of processes geared to understanding and addressing the causes of maternal deaths in low- and middle-income countries.

First, there are two words in the concept, ‘social autopsy’–‘social’ and ‘autopsy’. According to the Merriam-Webster dictionary, social means *“of or relating to human society”* [12] and an autopsy is “*an examination of a body after death to determine the cause of death…*” [13]. Thus, a social autopsy is an exploration of the cause of death related to human society. Without overly complicating the issue, a social autopsy is simply an enquiry to unravel social reasons that contributed to a death. Whatever researchers choose to do with the data collected – create community awareness, facilitate community dialogue, or inform policy – may be part of a broader community maternal and perinatal death surveillance and response (MPDSR) process, but these activities do not fall under the umbrella of an enquiry into the human society-related causes of death, and as such are not part of the social autopsy (Fig 1).

Second, while we recognise the value of capturing implementation lessons as was done in the recent global report by the WHO [14], we argue that this is only a first step. There is an urgent need for a harmonised guide for social autopsies led by the WHO, as has been done for verbal autopsy [10]. This guide should be based on strong methodological underpinnings and experiences of researchers who have conducted social autopsies. Some lessons from those experiences include the psychosocial stress that family members and neighbours face in being interviewed about the deceased, with some saying, *“…it hurts me afresh”* despite recognising the benefit of the enquiry [15], the value of qualitative data, despite its collection being labour intensive, and concerns raised about the length of some existing tools [11]. In our view, social autopsies should rely more on qualitative data, conducted with robust psychosocial support accessible to participants, and if linked to a verbal autopsy, be conducted immediately afterwards, thereby reducing cost that might be associated with repeated participant engagements.

Third, we argue that social autopsies need to be analysed at a scale small enough to generate valuable and homogenous community insights while being reported at a scale large enough to ensure anonymity. It is not unlikely that a social autopsy report of the two or three maternal deaths that occurred in ‘Ward A’ in 2024 will be easily identifiable by people in the community. If the scale is small, the community’s name should be anonymised to safeguard relatives, neighbours, and health workers who might be deemed complicit in the maternal death. There is a reason why a similar enquiry process to social autopsies that is conducted in high-income countries is called ‘confidential’ enquiry [16].

In conclusion, there is no doubt that social autopsies offer rich insights that can inform actionable responses. However, specific methodological guidance is urgently needed and consideration given to its implementation to ensure that it is conducted in a way that its benefits outweigh the risks to participants, guarantees their anonymity and confidentiality, and delivers good value-for-money. As the field moves forward, it is important to keep in mind that it was someone’s spouse, daughter, mother or sibling who died, and while we seek knowledge for action through social autopsies, the deceased still deserve dignity in death.
